# MaSS-Droid: Android Malware Detection Framework Using Multi-Layer Feature Screening and Stacking Integration

**DOI:** 10.3390/e27121252

**Published:** 2025-12-11

**Authors:** Zihao Zhang, Qiang Han, Zhichao Shi

**Affiliations:** 1 School of Computer Science and Engineering, North Minzu University, Yinchuan 750021, China; 2 Key Laboratory of Intelligent Image and Graphic Processing of State Ethnic Affairs Commission, North Minzu University, Yinchuan 750021, China

**Keywords:** Android malware detection, static analysis, feature selection, Stacking integration

## Abstract

In recent years, the frequent emergence of Android malware has posed a significant threat to user security. The redundancy of features in malicious software samples and the instability of individual model performance have also introduced numerous challenges to malware detection. To address these issues, this paper proposes a malware detection framework named **Mass-Droid**, based on **M**ulti-feature **a**nd Multi-layer **S**creening for adaptive **S**tacking integration. First, three types of features are extracted from APK files: permission features, API call features, and opcode sequences. Then, a three-layer feature screening mechanism is designed to effectively eliminate feature redundancy, improve detection accuracy, and reduce the computational complexity of the model. To tackle the problem of high performance fluctuations and limited generalization ability in single models, this paper proposes an adaptive Stacking integration method (Adaptive-Stacking). By dynamically adjusting the weights of base classifiers, this method significantly enhances the stability and generalization performance of the ensemble model when dealing with complex and diverse malware samples. The experimental results demonstrate that the MaSS-Droid framework can effectively mitigate overfitting, improve the model’s generalization capability, reduce feature redundancy, and significantly enhance the overall stability and accuracy of malware detection.

## 1. Introduction

With the rapid development of information technology, smartphones have become an integral component of modern life; these devices are ubiquitously utilized [1]. The Android operating system, leveraging its openness and extensive application ecosystem, dominates billions of smartphones and tablets worldwide and commands nearly 70.1% of the global market share, supporting approximately 4 billion active users across 190 countries [2]. However, the openness of the Android ecosystem and its massive user base have also made it a primary target for malicious entities [3]. Consequently, it poses substantial threats to user information security and property safety [4]. Traditional malware detection methods often rely on single-feature-based selection, which frequently results in suboptimal performance against novel malware variants. While conventional machine learning approaches demonstrate proficiency in pattern recognition, their generalization capability markedly deteriorates when handling high-dimensional features, thereby compromising detection efficiency and accuracy.

Android malware analysis techniques are classified as static, dynamic, or hybrid [5]. Yan et al. [6] proposed a gradient-based attack methodology for dynamic malware analysis by extracting API sequences during execution in sandbox environments. Slavisa et al. [7] expanded traditional feature sets through comprehensive behavioral reports generated by sandboxes and demonstrated superior performance compared to exclusively API sequence-based approaches. Aldhafferi [8] combined support vector regression with dynamic feature analysis to capture complex nonlinear relationships in application behaviors, achieving 95.74% detection accuracy, which outperformed conventional machine learning methods. Although dynamic analysis effectively mitigates emerging malware, it incurs resource-intensive monitoring and substantial computational overhead [9]. Consequently, static analysis is often favored when resource efficiency is critical, as it enables rapid analysis of application structure and code without execution. Yan et al. [10] developed a multi-dimensional feature fusion approach utilizing a dual-layer classification framework to address limitations of traditional methods in handling code obfuscation and data imbalance. The MSD method  [11] constructs heterogeneous graph features incorporating semantic and positional information through deep learning algorithms and API-based feature extraction to facilitate malware identification. Hemalatha  [12] transformed malware binaries into grayscale images by employing an enhanced DenseNet architecture integrated with reweighted class-balanced loss functions to enhance detection accuracy and model generalization while mitigating class imbalance. Therefore, efficient and accurate static analysis methods have attracted considerable research interest in both the academic and industrial domains.

Although a range of methods have been developed for Android malware detection, traditional static analysis approaches can be easily circumvented by simple code obfuscation techniques, thereby limiting their effectiveness in real-world scenarios. In contrast, dynamic detection techniques perform malware identification by monitoring various runtime characteristics, such as system calls, network traffic, battery usage, and application data flow during execution.

Ananya et al. [13] proposed a dynamic analysis method leveraging system call patterns to enhance classification performance, effectively mitigating the detection failures often encountered by static analysis due to code obfuscation. Furthermore, Mahindru et al. [14] developed a web-based dynamic analysis framework, attaining an accuracy of 98.8% in real-world malware detection scenarios.

Despite the significant improvements in detection accuracy offered by dynamic analysis approaches, these methods are generally associated with considerable overhead in terms of time and computational resources. Moreover, the reliability of dynamic detection results is often compromised by variations in the testing environment. Additionally, some sophisticated malware can actively detect the presence of analysis environments and conceal their malicious behaviors during monitoring, thereby evading detection.

Given these challenges, static analysis remains the predominant technique in practical applications due to its broader code coverage, lower resource consumption, and operational convenience. However, current static detection approaches for Android malware also face substantial limitations. Most notably, existing studies often rely on constrained feature representations, utilizing only a limited subset of potential feature dimensions. This narrow scope fails to adequately capture the complex and multi-dimensional characteristics of malware, resulting in impaired model efficacy—particularly when confronting novel or sophisticated threats. As a result, the accuracy of detection is compromised, and the ability to identify complex malicious behaviors is significantly diminished. Furthermore, many traditional static detection methods employ singular detection models, which inherently suffer from low robustness, suboptimal accuracy, and limited generalization capability. Collectively, these shortcomings present significant obstacles to the real-world deployment and effectiveness of conventional malware detection techniques.

The main challenges can be summarized as follows:

- **Limitations of single-feature approaches**: Extensive research and practice predominantly utilize single or highly restricted feature subsets. This single-dimensional feature extraction strategy introduces fundamental limitations within detection models. The behavioral patterns of malware are inherently complex and multi-dimensional. Focusing solely on a single feature is inherently insufficient to effectively characterize malicious behavior. This compromises detection efficacy and versatility, resulting in significant suboptimal performance particularly when detecting sophisticated and novel evasive threats.
- **Feature redundancy**: A pervasive issue of high feature correlation and duplication (termed feature redundancy) exists in malware samples. This not only increases model complexity but also impedes the learning of discriminative patterns, consequently diminishing detection accuracy. Hence, effective selection of salient features and dimensionality reduction are critically essential.
- **Limitation of single-model approaches**: Relying on a single detection model often induces convergence to local optima during training, inducing performance fluctuations and limited generalization capability. When processing polymorphic malware, it exhibits substantial instability. Developing methods to integrate multiple base learners and construct robust detection frameworks constitutes a critical research direction requiring urgent investigation.

This study proposes MaSS-Droid, a malware detection framework leveraging multi-layer feature screening and adaptive Stacking integration. Specifically, three types of features are extracted from APK files: permission features, API call features, and opcode sequences. Employing multi-layer screening, features are refined to generate a discriminative feature subset. The existing static analysis methods are summarized, and an adaptive Stacking integration method is proposed to improve the accuracy and efficiency of detection. A multi-classifier ensemble model is formulated to optimize detection robustness.Unlike many existing Android malware detection approaches that rely on a single feature type, a shallow feature reduction step, or a fixed-weight ensemble, MaSS-Droid jointly integrates a three-stage multi-feature screening pipeline with an adaptive-weight stacking mechanism. To the best of our knowledge, this combination of multi-layer feature screening and adaptive-weight ensemble learning has not been systematically explored in prior Android malware detection research, which highlights the methodological novelty of our framework. The principal contributions of this study are summarized as follows:

- A novel framework integrating multi-feature selection and ensemble detection is devised. To overcome the limitation of single-feature representation in static detection, the MaSS-Droid framework is developed. This framework synthesizes permission features, API call graphs, and opcode sequences into a unified feature representation. This architecture enables comprehensive characterization of malware’s multi-dimensional behaviors through multi-view feature fusion, substantially improving detection capability against novel and variant malware.
- We devise a three-stage feature screening mechanism. Stage 1 applies variance thresholding to eliminate low-variance features below a predefined threshold. Stage 2 evaluates feature discriminability, discarding features exhibiting insignificant distributional divergence between malware and benign samples. Stage 3 leverages information gain to filter low-importance features. This mechanism minimizes dimensionality while retaining discriminative signatures, reducing computational overhead.
- In response to the significant performance fluctuations in and insufficient generalization ability of the single detection model, this paper proposes Stacking integration based on adaptive weight allocation. This framework integrates the decision advantages of heterogeneous base classifiers and combines adaptive dynamic adjustment of model weights, significantly enhancing the overall detection stability and generalization ability.

The structure of the remaining part of this article is as follows: Section 2 reviews the previous works related to this study; Section 3 elaborates on the proposed methods and related algorithms; Section 4 introduces the evaluation metrics and the used datasets; Section 5 evaluates the proposed model based on real datasets through experiments; Section 6 summarizes the advantages and limitations of the model and proposes the next work plan.

## 2. Related Work

In recent years, in the field of computer security, numerous researchers have conducted extensive experimental studies on the issue of malware detection and have achieved remarkable results from multiple perspectives. This section will focus on the three challenges mentioned in the previous section and highlight the latest advancements made by scholars in recent years. Arif et al. [15] proposed an optimization technique to identify optimal features combined with machine learning algorithms for malware detection. The SIGPID framework [16] enhances Android malware detection efficiency and accuracy by mining permission data and employing multi-layer data pruning to filter critical permissions. Pathak et al. [17] developed an Android malware detection model by extracting essential permission features and implementing machine learning algorithms such as Random Forest. PermPair [18] analyzes permission-pair relationships in Android applications, constructs malicious-benign graphs, and utilizes multi-objective optimization to improve detection accuracy, ultimately achieving an efficient static malware detection method.

Pektas et al. [19] introduced an Android malware detection approach centered on opcode sequence features, generating sequences through instruction call graphs of all execution paths and classifying them with deep learning models to significantly enhance detection precision. The APILI framework [20], based on dual attention mechanisms, automatically locates underlying API calls during dynamic execution trajectories of malware, substantially improving malicious behavior detection and interpretation accuracy. Azar et al. [21] proposed MIFIBoost, integrating mutual information filtering with XGBoost feature reordering to achieve efficient and robust Android malware detection using static byte n-gram features.

Bhat [22] devised a multi-stage feature selection method based on feature discrimination and information gain filtering, which progressively compresses the static feature space to improve Android malware detection accuracy, achieving 96.28% highest accuracy with Random Forest classifiers. AIJarrah et al. [23] presented a detection method combining contextual features, API calls, and permission information optimized through information gain filtering, attaining 99.4% detection accuracy with Random Forest. Yoo et al. [24] proposed AI-HydRa, a hybrid decision model that integrates random forest and deep learning models to enhance malware detection capabilities. Zhu et al. [25] proposed a hybrid deep network learning framework called SHLMD, which utilized stacked hybrid learning MSAE and SDAE for detecting malware.

Current research in Android malware detection faces significant challenges at both the model and feature levels. At the model level, while ensemble learning is widely recognized for improving performance, many existing methods remain constrained by homogeneous base learners or rigid fusion strategies. This limits their ability to fully leverage the complementary strengths of heterogeneous models, resulting in insufficient generalization capability and stability when confronted with complex and evolving malware variants. Furthermore, while deep learning models offer powerful automatic feature representation, they often introduce substantial computational costs, complex hyperparameter tuning, and a lack of model interpretability, posing significant barriers to their deployment in resource constrained or transparency critical scenarios. In parallel, at the feature level, the effective distillation of highly discriminative feature subsets from high-dimensional and redundant raw data remains a critical, unresolved issue, undermining the robustness of detection systems.

To address these dual challenges, this paper proposes MaSS-Droid, a novel detection framework designed to balance high performance with practical utility. The framework first incorporates a multi-layer feature screening mechanism that systematically refines fused permission, API call, and opcode features through successive stages of variance thresholding, discriminative power analysis, and frequency-adjusted information gain. This process effectively mitigates feature redundancy and preserves the most salient attributes. Subsequently, we introduce an adaptive Stacking integration model that strategically employs a diverse set of traditional machine learning algorithms as base learners. This design intentionally prioritizes computational efficiency and model interpretability, attributes that are often compromised by deep learning, while dynamically leveraging the complementary decision boundaries of the constituent models. Instead of introducing another deep neural model, MaSS-Droid adopts an adaptive stacking strategy with traditional yet heterogeneous base learners, aiming to achieve a better balance between accuracy, robustness, interpretability, and deployment feasibility on resource-constrained devices.Consequently, MaSS-Droid achieves high detection accuracy and enhanced robustness without incurring the prohibitive overhead associated with deep neural networks, thereby presenting a potent and deployable solution for static Android malware analysis.

## 3. Materials and Methods

### 3.1. Methodology Framework

**Figure 1:**
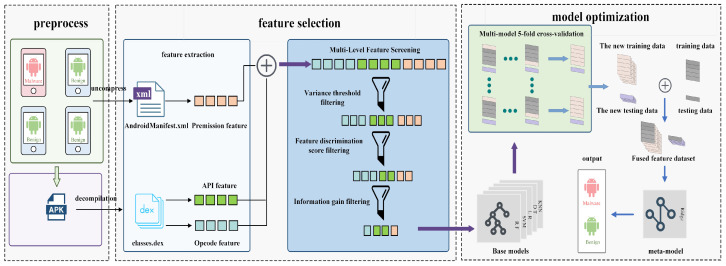
Method Diagram.

This paper presents a malicious software detection framework based on static analysis. By conducting static analysis on APK files, this framework constructs the overall architecture as illustrated in Figure 1. The framework comprises three key components:

- **Feature Extraction**: Multiple feature sets are extracted from the APK files, encompassing permissions, API calls, and opcode sequences. Each feature set captures distinct aspects of the Android application, aiming to reveal potential indicators of malicious behavior.
- **Data Processing**: Data Reading and Preparation: The feature set files for permissions, API calls, and opcodes are first read. The data undergoes cleaning, standardization, and merging to ensure consistency and integrity. Feature Selection: To reduce dimensionality, mitigate feature redundancy and computational burden, while preserving critical discriminative information, feature selection is performed using three distinct metrics: (i) Variance Threshold: Filters out low-variance features. (ii) Feature Discrimination Score: Selects features based on their ability to distinguish between classes. and (iii) Prioritizes features based on the reduction in entropy/uncertainty they provide for classification. This process retains the most discriminative features crucial for effective malware detection.
- **Optimization of Ensemble Learning**: A two-layer heterogeneous ensemble is established by first training five diverse base learners, including Support Vector Machine (SVM), k-Nearest Neighbors (KNN), Decision Tree (DT), Random Forest (RF), and Logistic Regression (LR), using five-fold cross-validation to generate meta-features. Each base model’s weight is adaptively assigned based on its validation F1-score, such that more reliable classifiers contribute proportionally to the final decision. Subsequently, a Ridge Classifier meta-learner is trained using these weighted meta-features to determine the optimal combination strategy. Consequently, the overall detection performance is enhanced.

### 3.2. Feature Extraction

**Figure 2:**
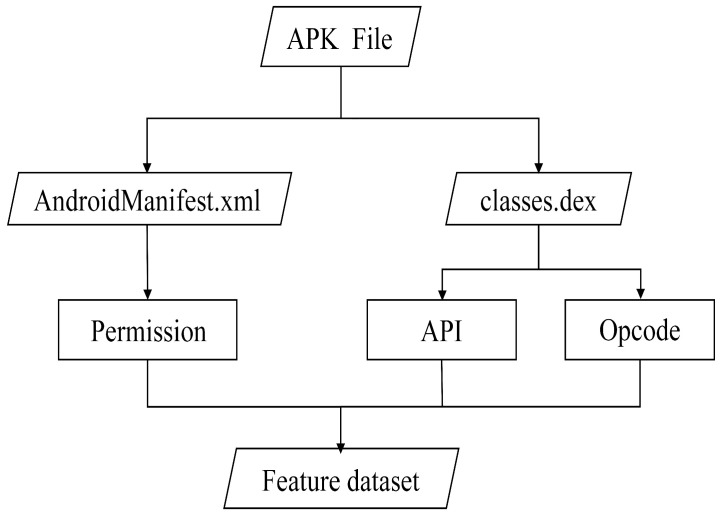
Static analysis process.

APK samples are processed using decompilation tools. Subsequently, three core feature types—permission features, API features, and opcode features—are extracted based on static analysis. A feature matrix, illustrated in Figure 2, is constructed where rows represent applications and columns represent features. Cell values indicate the presence status of the corresponding feature within the respective application. The output is saved as a structured .csv file for use by machine learning models.

**Figure 3:**
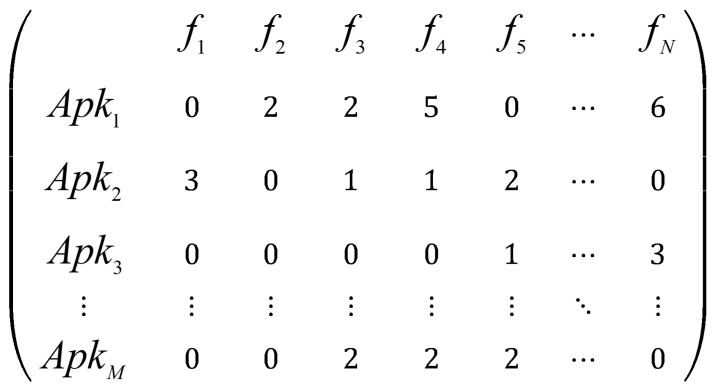
Feature matrix.

In recent years, researchers have proposed numerous methods for Android malware static detection. These methods are primarily categorized based on the features they extract: permission-based, API call-based, and opcode-based. Regardless of the specific features intended for extraction, the primary workflow for Android malware static analysis unfolds from the APK file, as shown in Figure 3. This workflow involves either extracting permission features from the AndroidManifest.xml file, extracting API calls from the classes.dex file, or integrating both types of features into the dataset.

### 3.3. Data Processing

#### 3.3.1. Preprocessing

In malware detection, feature preprocessing is a critical step in the data preparation process, aimed at enhancing model accuracy and efficiency. Common malware features include file API calls, system calls, permissions, behavior logs, and others. These features typically undergo the following preprocessing steps:

(i) Data Cleaning: This step handles missing values, outliers, and noisy data to ensure data quality. Common methods include interpolation-based imputation, removing samples with missing values, or employing suitable models to estimate missing values.

(ii) Data Binarization: Machine learning datasets often comprise multiple features with varying value ranges, which can degrade model training performance. To make the influence of different features more comparable, numerical features are frequently subjected to binarization. Binarization typically transforms data into a distribution of 0 or 1, where values exceeding a specified threshold are converted to 1, and values equal to or below the threshold are converted to 0.

**Figure 4:**
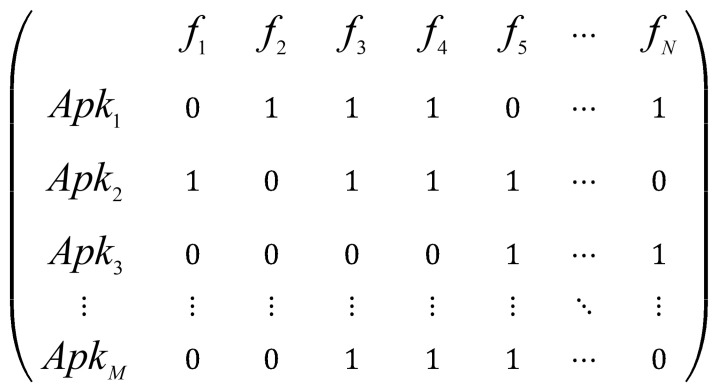
Processed matrix.

The feature matrix resulting from data cleaning and binarization preprocessing is illustrated in Figure 4.

#### 3.3.2. Multi-Level Feature Screening

The proposed multi-level feature screening method comprises three steps of feature reduction, designed to eliminate non-informative features.

Firstly, at the first stage of feature screening, features with low variance below a set threshold are removed using the variance threshold method. Features exhibiting low variance typically carry less information and can therefore be discarded. Secondly, in the second stage, features are evaluated for their effectiveness in discriminating between the two classes of software (malicious and benign). This step removes features that occur with similar frequency in both malicious and benign files, as such features lack sufficient discriminatory power for classification tasks. Finally, to further enhance classification performance, the method applies Information Gain to remove less important features. This strategic stepwise reduction in features eliminates redundant and insignificant ones, which are then utilized with machine learning methods to build an efficient malware detection system.

##### Variance Threshold

To enhance model performance and computational efficiency, this study first applies variance thresholding to preprocess the dataset and remove low-variance features. Such features exhibit identical or highly similar values across the vast majority of samples, providing limited discriminative information for classification tasks. In machine learning models, retaining these features not only fails to improve prediction accuracy but also increases computational complexity, introduces potential noise, and may even impair the model’s generalization capability. Although certain low-variance features might retain contextual relevance in specific scenarios, their discriminatory value remains limited in most classification contexts. Consequently, the removal of low-variance features has become a standard preprocessing step in feature selection and has been widely adopted across various domains such as image recognition, text classification, and bioinformatics. It calculates the variance of each feature and applies a threshold to filter out features exhibiting low variance below this set value. For a given feature dataset comprising *M* samples and *N* features, the mean value $\mu_{j}$ for a specific feature $f_{j}$ can be calculated as follows:

(1) $$\mu_{j}=\frac{1}{M}\sum_{i=1}^{M}x_{ij}$$

where $x_{ij}$ refers to the *i* sample in a certain feature $f_{j}$. The variance of this feature is calculated as:

(2) $$\mathrm{Var}\left(f_{j}\right)=\frac{1}{M}\sum_{i=1}^{M}{\left(x_{ij}-\mu_{j}\right)}^{2}$$

We employ variance thresholding as an initial feature screening step. If the variance of a certain feature $Var(f_{j})$ is less than the set threshold θ ($Var(f_{j})$<θ), then it is considered that the feature has changed too little and can be removed. The remaining features then proceed to a subsequent round of screening.

##### Feature Discrimination Score

This method filters out features that fail to distinguish between malware and benign samples. Features are scored based on their distribution patterns across the two classes. A higher score is assigned to features that predominantly occur in one class, while a lower score indicates similar occurrence rates in both classes. Low-scoring features are removed as they lack discriminative power. The score is calculated as follows:

(3) $$\mathrm{Score}\left(f_{j}\right)=\frac{|f_{j}^{b}-f_{j}^{m}|}{\max(f_{j}^{b},f_{j}^{m})}$$

where:

- $f_{j}^{b}$: Frequency of feature *j* in benign files,
- $f_{j}^{m}$: Frequency of feature *j* in malicious files.

If the score of a certain feature $Score(f_{j})$ is less than the set threshold δ ($Score(f_{j})$<δ), then it is considered that the feature has littler discriminative capability and can be removed. The remaining features then proceed to a subsequent round of screening.

##### Frequency Adjusted Information Gain

Entropy H(Y) quantifies the uncertainty of class labels without feature information:

(4) $$H\left(Y\right)=-\sum_{k=1}^{K}p_{k}\log_{2}p_{k}$$

Here *K* represents the category of *Y*, in this case *K* = 2 (benign and malware), and $p_{k}$ the probability of occurrence of the class.

This paper employs information entropy to quantify the uncertainty of classification events. Conceptually, information entropy represents the potential information content contained in the classification outcome *Y*. A higher information entropy indicates greater information gain from the classification process. When an event is known to be impossible (probability = 0), the information gain is zero. The maximum information entropy is achieved when events occur with equal (probability = 0.5). Therefore, for malware classification, the maximum information entropy—and consequently the greatest information gain—is obtained only when malicious and benign software samples are present in equal quantities.

To enhance the effectiveness and representativeness of feature selection, this paper proposes an improved information gain scoring mechanism based on traditional feature screening methods, which incorporates feature occurrence frequency [26,27,28]. This approach not only considers a feature’s contribution to reducing class label entropy but also accounts for its frequency in the sample set. This dual consideration significantly improves the stability of the final feature subset and enhances practical detection performance.

The conditional entropy H(Y|X), which measures the uncertainty of the class label given the presence $\left(f_{j}=1\right)$ or absence $\left(f_{j}=0\right)$ of feature $f_{j}$, is calculated as follows:

(5) $$H\left(Y|f_{j}\right)=p\left(f_{j}=1\right)H\left(Y|f_{j}=1\right)+p\left(f_{j}=0\right)H\left(Y|f_{j}=0\right)$$

Traditional information gain $\mathrm{IG}(f_{j})$ is defined as:

(6) $$\mathrm{IG}\left(f_{j}\right)=H\left(Y\right)-H\left(Y|f_{j}\right)$$

However, the traditional information gain calculation method does not consider the feature’s occurrence frequency in the sample set, potentially leading to overestimation of rare features (low occurrence frequency) due to chance. Therefore, this paper further introduces the feature occurrence frequency $F(f_{j})$ as an adjustment factor to correct the bias in feature evaluation.

To enhance stability and detection performance, we introduce **F**requency **A**djusted **I**nformation **G**ain (FAIG). This method incorporates feature density $F(f_{j})$ to mitigate overestimation of rare features, then the $F(f_{j})$ is same with $p(f_{j}=1)$ of Equation (5):

(7) $$F\left(f_{j}\right)=\frac{Count(f_{j}=1)}{M}$$

where $Count(f_{j}=1)$ is the number of samples containing feature $f_{j}$, i.e., the number of presence of feature $f_{j}$, and *M* denotes the number of samples.

Integrating information gain with feature density, the FAIG is defined as

(8) $$FAIG\left(f_{j}\right)=F\left(f_{j}\right)\cdot \alpha \cdot IG\left(f_{j}\right)$$

where α is the density impact adjustment parameter, used to control the degree of influence of feature occurrence frequency on the final score. Through the frequency weighting mechanism, the scores of rare features (low density) are moderately suppressed, while the scores of key features with moderate occurrence frequency in the samples are reasonably enhanced. Finally, all candidate features are sorted in descending order according to their $FAIG(f_{j})$ scores, and the top-ranked features are selected for subsequent use.

### 3.4. Adaptive Stacking Integration

In the context of malware detection, it has been widely observed that conventional single-model architectures are prone to converging to local optima during the training phase, which consequently leads to sub-optimal generalization performance and unsatisfactory detection accuracy. To systematically address this limitation, the present work adopts a model-fusion perspective grounded in ensemble-learning theory.

Specifically, multiple high-capacity classifiers are integrated through a Stacking strategy, and an adaptive mechanism is introduced to dynamically re-weight the contributions of individual base learners, thereby mitigating the instability and weak generalization exhibited by any single model when confronted with complex and heterogeneous malware instances.

Stacking is an ensemble learning method whose core idea is to fuse the predictive capabilities of multiple base learners through hierarchical modeling [29,30]. Unlike traditional voting or averaging strategies, Stacking introduces a meta-learner to learn how to optimally combine the outputs of base models [31,32]. The advantage of this approach lies in its ability to fully leverage the respective discriminative strengths of different types of classifiers, enhance the effectiveness and classification accuracy of the overall heterogeneous classifier model, and reduce variance and bias. This paper proposes a Stacking integration framework based on adaptive weight allocation. This framework integrates the decision advantages of heterogeneous base classifiers and combines the adaptive dynamic adjustment of model weights.

**Figure 5:**
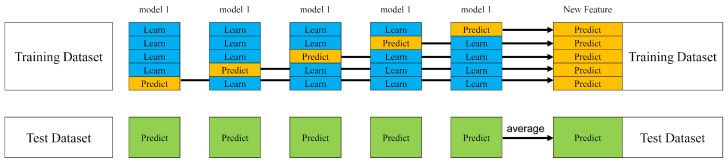
Single-model stacking.

The Stacking ensemble used is illustrated in Figure 5 (Schematic diagram of single-base-model Stacking). Its core process involves the base model performing strict 5-fold cross-validation on the original training set. In each fold of validation, the model is trained using four folds of data and makes predictions on the held-out fold. After iterating through all five folds, a complete set of predictions for the entire original training set is ultimately obtained. These predictions are not the final output; instead, they are constructed into a new type of meta-feature. Specifically, they are appended as a new attribute column to the original training set, forming a secondary training set. Subsequently, a meta-model is trained on this secondary training set to learn how to optimally combine the predictive information from the base model. For the test set, the trained base model is used directly to make predictions. These predicted values are then fed as new features into the already trained meta-model, which finally produces the ensemble prediction result.

**Figure 6:**
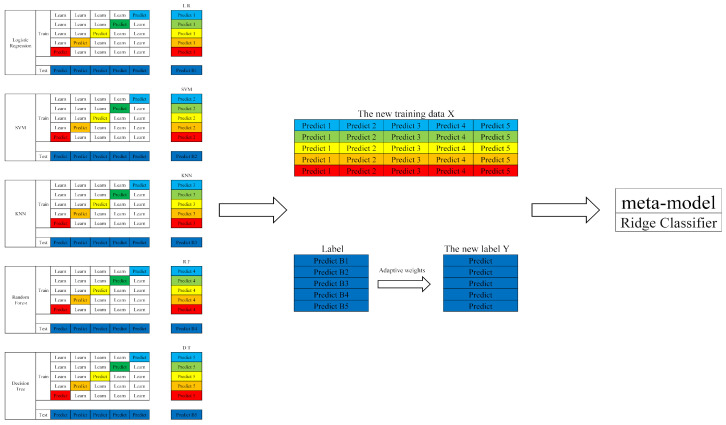
Multi-model stacking.

Nevertheless, employing Stacking with only a single base model provides limited model diversity, which restricts the ensemble’s ability to generalize across complex or heterogeneous data distributions. In order to fully exploit the complementary strengths of multiple learning algorithms and enhance the ensemble’s overall predictive power, we propose an advanced stacking ensemble method featuring adaptive weight allocation. The schematic diagram of multi-model Stacking is shown in Figure 6.

In this study, we employ five representative and heterogeneous classifiers as base learners: Support Vector Machine (SVM), K-Nearest Neighbors (KNN), Decision Tree (DT), Random Forest (RF), and Logistic Regression (LR). This diverse set of models, including linear, large-margin, distance-based, and tree-based classifiers, is deliberately selected to maximize the diversity of decision boundaries, thereby promoting error decorrelation among the base learners. Each base classifier is trained independently on the training folds and performs standard 5-fold cross-validation to generate out-of-fold (OOF) predictions. These OOF predictions from each classifier serve as classifier-specific meta-features. By concatenating the meta-features from all base learners, we create a compact and information-rich secondary training set that captures multiple perspectives of the original input data.

As the meta-learner, we adopt Ridge regression (a form of linear regression with L2 regularization). There are three key reasons for this choice: (1) OOF meta-features tend to be correlated across base learners, and L2 regularization helps mitigate multicollinearity and reduces the risk of overfitting on the typically small meta-level dataset; (2) Ridge regression is computationally inexpensive, making it suitable for deployment in resource-constrained environments; and (3) in small-sample meta-settings, a low-capacity, regularized linear model tends to be more stable and generalizable than highly expressive non-linear models. All base models and the meta-learner are trained using the same training split policy. The meta-learner is trained on the concatenated OOF vectors and evaluated on held-out test folds to prevent information leakage.

This approach enables the stacking ensemble to fully leverage the complementary strengths and predictive differences among the diverse base models. By effectively exploiting this complementarity, the ensemble significantly enhances the overall model’s robustness and predictive accuracy. To optimize performance, key hyperparameters—including base model selection, meta-learner choice, and cross-validation strategy—must be systematically tuned using methods such as grid search or random search. This optimization is critical for preventing overfitting and ensuring model stability and generalization capability.

Within the Adaptive-Stacking framework, the first layer comprises a set of base learners responsible for producing initial probabilistic predictions, while the second layer consists of a meta-learner whose role is to aggregate and synthesize the outputs of the base learners into a final, calibrated decision.

To ensure an unbiased and efficient training pipeline, the dataset is first partitioned into a training set $D_{\mathrm{train}}$ and a test set $D_{\mathrm{test}}$. Direct utilization of identical folds for both base-learner and meta-learner training may induce overfitting due to data redundancy; hence, a 5-fold cross-validation protocol is employed over $D_{\mathrm{train}}$ to generate reliable predictions.

First, the training feature dataset $D_{\mathrm{train}}$ is defined as

(9) $$D_{\mathrm{train}}=\left\{\left(x_{1},x_{2},\ldots ,x_{j},y_{i}\right)|j=1,2,\ldots ,N,\;i=1,2,\ldots ,70\%*M\right\}$$

Here $x_{j}$ denotes the *j* th feature value and $y_{i}$ the *i* th sample. Meanwhile, *N* represents the number of selected features, and 70%∗M represents the number of training samples, which is 70% of the total number of samples *M*, i.e., the total number of samples was splitted into $D_{\mathrm{train}}$ (70%), therefore the rest samples is $D_{\mathrm{test}}$ (30%).

Subsequently, $D_{\mathrm{train}}$ is randomly partitioned into five mutually exclusive and equally sized subsets

(10) $$\left\{\begin{array}{c} D_{\mathrm{train}}=D_{1}\cup D_{2}\cup D_{3}\cup D_{4}\cup D_{5}\\ D_{p}\cap D_{q}=⌀\;\left(p\neq q\right) \end{array}\right.$$

Such that during the *p*-th cross-validation fold, subset $D_{p}$ is designated as the validation set $D_{\mathrm{valid}(p)}$, and the union of the remaining subsets $D_{\mathrm{train}}\setminus D_{p}$ serves as the training set $D_{\mathrm{train}(p)}$. This procedure guarantees that each base learner undergoes five independent training and validation cycles, thereby effectively mitigating the risk of overfitting.

Following the Stacking paradigm, five structurally diverse yet high-performance models are selected as first-layer base learners, formally denoted as $LM_{1},LM_{2},LM_{3},LM_{4},LM_{5}$, while a meta-learner LM with strong aggregation capacity—implemented here as RidgeClassifier—is appointed to the second layer. The detailed procedure is as follows. Equations (9)–(15) follow the formalism established in Reference [33].

For each fold p=1,…,5 the following partitions are constructed:

(11) $$\left\{\begin{array}{c} D_{\mathrm{train}(p)}=D_{\mathrm{train}}\setminus D_{p}\\ D_{\mathrm{valid}(p)}=D_{p} \end{array}\right.$$

Training base learner $LM_{u}$ on $D_{\mathrm{train}(p)}$ yields the parameter set

(12) $$P_{up}=LM_{u}\;(D_{\mathrm{train}(p)}),p=1,\ldots ,5,\;u=1,\ldots ,5,$$

Subsequently, a systematic test was conducted by each trained base learner $LM_{u}$ on the validation set $D_{\mathrm{valid}(p)}$, thereby obtaining the new training dataset  $D_{{\mathrm{train}}_{up}}^{*}$, which is defined as follows:

(13) $$D_{{\mathrm{train}}_{up}}^{*}=P_{up}\;\left(D_{\mathrm{valid}(p)}\right)$$

Subsequently, these resulting training sets $D_{{\mathrm{train}}_{up}}^{*}$ will be integrated to form a new training set, which will be used for the training of the next layer of meta-learners.

(14) $$D_{\mathrm{train}}^{*}=\left[\;{\left[D_{{\mathrm{train}}_{11}}^{*},\ldots ,D_{{\mathrm{train}}_{15}}^{*}\right]}^{⊤},\ldots ,{\left[D_{{\mathrm{train}}_{51}}^{*},\ldots ,D_{{\mathrm{train}}_{55}}^{*}\right]}^{⊤}\right]$$

Similarly, each trained base learner $LM_{u}$ is applied to the test set. Since they are trained on distinct subsets, their parameters diverge, leading to varied predictions on the same test set $D_{test}$. The output predictions from each base learner, based on their respective learned parameters, can be expressed as follows:

(15) $$D_{{\mathrm{test}}_{up}}^{*}=P_{up}\left(D_{\mathrm{test}}\right)$$

(16) $$D_{{\mathrm{test}}_{u}}^{*}=\frac{1}{5}\sum_{p=1}^{5}D_{{\mathrm{test}}_{up}}^{*},\;u=1,\ldots ,5.$$

For each trained base learner $LM_{u}$, the F1-score $\left(F1_{up}\right)$ can be computed on the validation folds $\left(D_{{\mathrm{test}}_{up}}^{*}\right)$ is employed as the performance criterion with the corresponding parameter set $\left(P_{up}\right)$. The normalized adaptive weight $\beta_{u}$ is then derived as

(17) $$\beta_{u}=\frac{\sum_{p=1}^{5}{F1}_{up}}{\sum_{v=1}^{5}\sum_{p=1}^{5}{F1}_{vp}},\;\sum_{u=1}^{5}\beta_{u}=1,$$

Here ${F1}_{up}$ or ${F1}_{vp}$ denotes the F1-score achieved by the *u*th or *v*th base learner on the validation data.

Sum the test set prediction results of each base learner according to the adaptive weights $\beta_{u}$, and generate the input for the meta-learner:

(18) $${Test}_{u}=\beta_{u}\cdot D_{{\mathrm{test}}_{u}}^{*}$$

Next, the new test set ${Test}_{new}$ for the meta-learner is defined as:

(19) $$Test_{new}=\left\{\begin{array}{c} Test_{1},Test_{2},Test_{3},Test_{4},Test_{5} \end{array}\right\}$$

Finally, the meta-learner is trained using the new training set $D_{train}^{*}$, and the learning parameter $P^{\prime }$ of the meta-learner is obtained as:

(20) $$P^{\prime }=LM\left(D_{train}^{*}\right)$$

Through the obtained trained meta-learner to detect the new test set ${Test}_{new}$, the final detection result *R* is obtained as follows

(21) $$R=P^{\prime }\left(Test_{new}\right)$$

### 3.5. Algorithm Analysis

**Figure 7:**
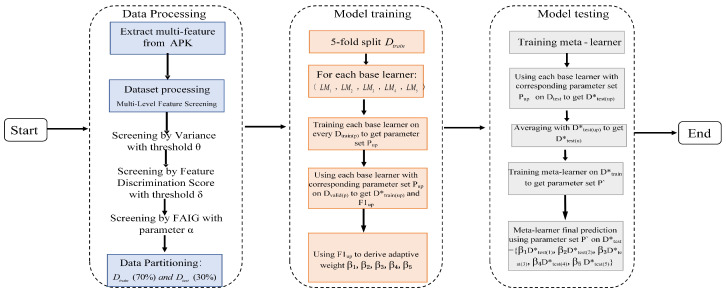
Flowchart of adaptive Stacking algorithm.

The proposed framework’s three-layer feature screening (variance threshold, discriminative power analysis, frequency-adjusted information gain) progressively refines multi-source fused permission, API, and opcode features. It eliminates ineffective low-variance features, subsequently discards features exhibiting low class separability, and ultimately identifies high-value features via FAIG, thereby ensuring input quality while reducing noise and dimensionality. The adaptive stacking strategy is integrated, replacing equal-weight fusion; dynamically computing normalized weights based on heterogeneous base classifiers 5-fold cross-validated F1-scores. This synergistic dual-optimization approach constructs a highly discriminative and generalizable malware detection framework. It substantially enhances detection accuracy while preserving consistent adaptability and robustness during complex APK analysis. The implementation flowchart is illustrated in Figure 7, and the corresponding algorithmic steps are detailed in Algorithm 1, which are isomorphic to the Equations (9)–(21) presented above, at the semantic level.
**Algorithm 1** Malware detection algorithm based on multi-feature screening and Adaptive Stacking  1:  **Input:** APK sample set $Y=\{\left(x_{1},x_{2},\ldots ,x_{j},y_{i}\right)|j=1,2,\ldots ,N,\;i=1,2,\ldots ,M\}$; Feature extraction methods (Permissions, API calls, Opcodes); Base learners $\left\{LM_{1},\ldots ,LM_{5}\right\}$; Meta-learner $LM_{meta}$; Threshold θ,δ; Density impact adjustment parameter α;  2:  **Output:** Detection results *R*  3:  **for** each APK sample $x_{i}$ in *Y* **do**  4:        Extract permission, API call, and opcode features, and concatenate as feature vector $x_{i}$  5:  **end for**  6:  Construct feature matrix *X*  7:  // Step 1: Variance threshold screening (Equations (1) and (2))  8:  **for** each feature $f_{j}$ in *X* **do**  9:        Compute variance $Var(f_{j})$(Equation (2))10:         **if** $Var(f_{j})<\theta$ **then**11:            Remove $f_{j}$ from *X*12:        **end if**13:  **end for**14:  // Step 2: Feature discrimination score (Equation (3))15:  **for** each feature $f_{j}$ in *X* **do**16:        Compute $f_{j}^{b}$ and $f_{j}^{m}$17:        Compute $Score(f_{j})$(Equation (3))18:        **if** $Score(f_{j})<\delta$ **then**19:            Remove $f_{j}$ from *X*20:        **end if**21:  **end for**22:  // Step 3: Frequency-adjusted information gain (Equations (4)–(8))23:  **for** each feature $f_{j}$ in *X* **do**24:        Compute information gain $IG(f_{j})$(Equation (6))25:        Compute feature frequency $F(f_{j})$(Equation (7))26:        Compute $FAIG\left(f_{j}\right)=F\left(f_{j}\right)\cdot \alpha \cdot IG\left(f_{j}\right)$(Equation (8))27:  **end for**28:  Rank all features by $FAIG(f_{j})$ in descending order and select the top *K* as $F_{\mathrm{selected}}$(Equation (8))29:  Split dataset into $D_{\mathrm{train}}$ (70%) and $D_{\mathrm{test}}$ (30%)30:  Partition $D_{\mathrm{train}}$ into 5 folds: $D_{\mathrm{train}}=D_{1}\cup D_{2}\cup D_{3}\cup D_{4}\cup D_{5}$31:  // Stacking training (Equations (9)–(14))32:  **for** each base learner $LM_{u}$, u=1,…,5 **do**33:        **for** p=1,…,5 **do**34:            Let $D_{\mathrm{valid}}\left(p\right)=D_{p}$, $D_{\mathrm{train}}\left(p\right)=D_{\mathrm{train}}\setminus D_{p}$(Equations (10) and (11))35:            Train $LM_{u}$ on $D_{\mathrm{train}}\left(p\right)$ to get parameter $P_{up}$(Equation (12))36:            Obtain a new training dataset $D_{\mathrm{valid}}\left(p\right)$ using $P_{up}$(Equations (13) and (14))37:            Compute F1-score $F1_{u}$ on $D_{\mathrm{valid}}\left(i\right)$38:        **end for**39:        Make output prediction $D_{{\mathrm{test}}_{u}}^{*}$ by learning the parameter $P_{up}$(Equations (15) and (16))40:  **end for**41:  // Adaptive weight calculation (Equation (17))42:  **for** each base learner $LM_{u}$ **do**43:        Compute normalized weight $\beta_{u}=\frac{\sum_{p=1}^{5}{F1}_{up}}{\sum_{v=1}^{5}\sum_{p=1}^{5}{F1}_{vp}}$(Equation (17))44:  **end for**45:  // Step 4: Testing ((18)–(21))46:  Obtain a new test dataset $Test_{\mathrm{new}}$ using $\beta_{u}$(Equations (18) and (19))47:  Obtain meta-learning parameter $P^{\prime }$(Equation (20))48:  Predict final results $R=P^{\prime }\left(Test_{new}\right)$(Equation (21))49:  **return** *R*


## 4. Experimental Setup

### 4.1. Evaluation Metrics

To evaluate the performance of our proposed method, we adopted a series of widely recognized evaluation metrics in the field of malware detection and machine learning. These metrics provide robust support for comprehensively measuring the model’s overall effectiveness, precision, and reliability. In the context of binary classification tasks, key terms include True Positive (TP), True Negative (TN), False Positive (FP), and False Negative (FN). For binary classification research, we commonly use Accuracy, Recall, Precision, and F1 Score as core indicators to evaluate model performance. These metrics not only ensure effective assessment of prediction accuracy and the performance of binary classification models but also fully consider the trade-off relationship between false positives and false negatives. Their specific mathematical definitions are as follows:

(22) $$Accuracy=\frac{TP+TN}{TP+TN+FP+FN}$$

(23) $$Precision=\frac{TP}{TP+FP}$$

(24) $$Recall=\frac{TP}{TP+FN}$$

(25) $$F1Score=\frac{2\times \mathrm{Precision}\times \mathrm{Recall}}{\mathrm{Precision}+\mathrm{Recall}}$$

In our evaluation framework, we adopted these four core metrics—Accuracy, Recall, Precision, and F1 Score—aiming to comprehensively and deeply understand the model’s performance. This multi-dimensional evaluation strategy ensures we can fully capture the strengths and potential limitations of the proposed detection framework, thereby providing a solid basis for subsequent model optimization and improvement.

### 4.2. Datasets

The dataset used in this experiment consists of the publicly available Drebin, AndroZoo, and CICMalDroid2020 datasets, as shown in Table 1, totaling 22,759 samples, including 15,906 malware samples and 6853 benign software samples. Specifically, the Drebin and AndroZoo datasets were used to validate the effectiveness of the proposed method.To ensure the reliability and reproducibility of the experimental results, the dataset was partitioned into training and test sets using stratified random sampling at a ratio of 7:3. This partitioning method not only ensured an adequate sample size in the training set for model training but also retained a sufficient number of test samples to evaluate the model’s generalization performance. To further enhance model validity and prevent overfitting, this study employed 5-fold cross-validation for model training and validation.

To validate the effectiveness of the proposed method for identifying new, previously unseen malware, we applied the trained model to the CICMalDroid2020 dataset. It is important to note that all hyperparameters in the method’s framework, including feature selection thresholds, were adjusted exclusively on the Drebin and AndroZoo datasets. The CICMalDroid2020 dataset was used only for external validation to assess the model’s generalization performance on new malware samples.

## 5. Results and Analysis

All experiments were conducted in a Python 3.8 environment, with code compiled and executed using PyCharm 2021.2. The tests were performed on a 64-bit Windows 10 operating system, with hardware configurations comprising an Intel(R) Xeon(R) Gold 6154 CPU and an NVIDIA TITAN V graphics card.

### 5.1. Threshold Selection

In the first layer of feature selection, variance thresholds were used to filter out irrelevant features. These thresholds were determined by comparing various values and analyzing the proportion of features retained at each threshold. The goal was to discard features that did not contribute meaningfully to the model’s predictive power, thereby reducing the feature space and improving the efficiency of subsequent layers.

**Figure 8:**
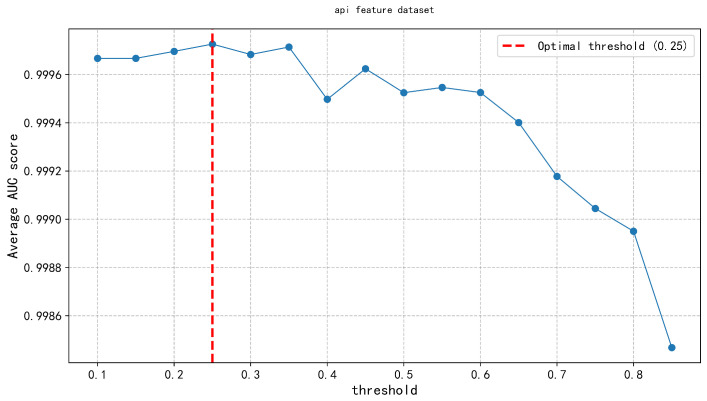
API threshold.

**Figure 9:**
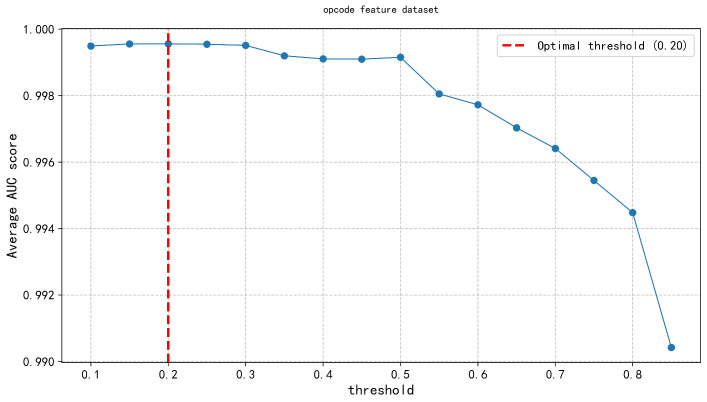
Opcode threshold.

**Figure 10:**
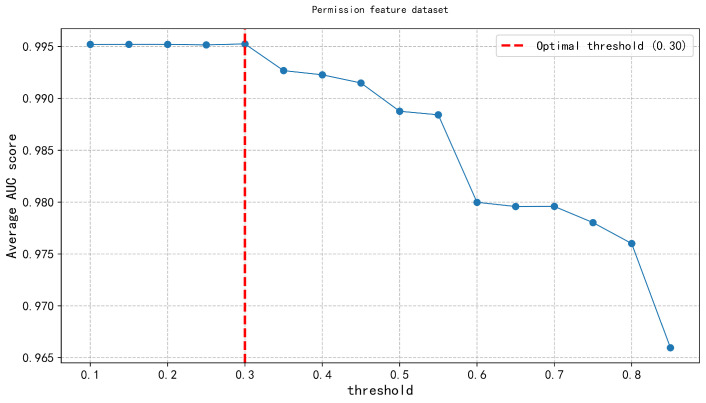
Permission threshold.

In the second layer, the Feature Discrimination Score was applied across three types of malware feature datasets (API calls, opcode features, and permission features). An exhaustive method was systematically applied to evaluate model performance under different thresholds, ranging from 0.1 to 0.9 in increments of 0.05. The results, shown in Figure 8, Figure 9 and Figure 10, indicate that the feature discrimination score threshold within the range of 0.20–0.30, where model performance peaks. Specifically, we determined the optimal threshold, as indicated by the vertical red dashed line in the figure. This threshold selection process effectively retained the most discriminative features, enabling accurate differentiation between malware and benign software samples, while significantly reducing computational complexity and improving detection efficiency.

### 5.2. Multi-Stage Screening

In this experimental study, we systematically evaluated the effectiveness of three key static features—API calls, opcodes, and permission information—for Android malware detection. To assess their robustness and optimal utility, each feature type was analyzed in two distinct states: (1) its original, raw form and (2) after undergoing a comprehensive multi-stage screening and processing pipeline designed to refine and select the most discriminatory attributes. This comparative evaluation was performed using five widely adopted and representative machine learning classifiers.

The results of these machine learning classifiers are shown in Table 2, Table 3 and Table 4. The results indicate that the multi-level filtering strategy has overall improved the classification performance of the model, bringing positive optimizations in most model and feature combinations. Especially in the API and permission features, the effect is the most significant. During the feature selection stage, a multi-level filtering processing mechanism was introduced, fully considering the occurrence frequency, key feature importance information, and information gain to measure the discriminatory ability of features in reducing the entropy of the category. By integrating these three factors, this paper achieved a dual assessment of the local discriminative ability of features and the global correlation strength, thereby effectively selecting more representative and generalizable key features and improving the overall performance of the malware detection model.

Specifically, LR benefited most significantly on API features, improving from 0.9905 with original features to 0.9955. This indicates that this model is relatively sensitive to redundant information when facing high-dimensional sparse features, and multi-stage screening effectively compresses ineffective features. SVM also performed excellently across features, suggesting its good adaptability to the screened high-quality feature sets. RF demonstrated stability and high robustness across all dimensions, achieving stable improvements on all three feature sets, validating the enhancing effect of IM screening.

In contrast, the performance of KNN was more complex: it showed improvement with API and opcode features but experienced a decrease in accuracy (from 0.9605 to 0.9571) with permission features. This might be because permission features themselves have extremely high dimensionality and sparse semantics. multi-stage screening might disrupt the feature space structure upon which KNN relies while compressing dimensions, causing its distance-based metric mechanism to fail. DT showed generally moderate performance, with slight decreases observed in some dimensions like opcodes, indicating its sensitivity to the structure after feature screening, leading to issues like overfitting or insufficient information utilization.

Furthermore, the overall gain for opcode features was less significant than for API and permission features. This is related to the relatively smaller number of opcode features and their limited representational capacity, which constrains the compression effect of multi-stage screening. Therefore, subsequent research could consider introducing deep learning techniques to embed and model opcode sequences to enhance their semantic expression capability.

### 5.3. Model Optimization

Among the ensemble methods evaluated, Adaptive Stacking demonstrates the highest performance, exhibiting superior accuracy, precision, recall, and F1-score. This indicates that Adaptive Stacking, by intelligently combining multiple base learners, is able to enhance prediction performance, particularly in terms of accuracy and recall. This method’s robust combination of diverse models ensures effective generalization across the dataset, achieving near-optimal results.

Following closely behind, Stacking and Voting methods show very similar performance metrics. Stacking, utilizing RidgeClassifier as a meta-model, marginally outperforms Voting, especially in recall. This advantage can be attributed to the meta-model’s ability to adaptively assign appropriate weights to each base learner’s prediction, thereby improving the model’s ability to handle imbalanced data. In contrast, the Voting method, while effective, lacks a mechanism to explicitly adjust for the differing performance levels of individual base models, leading to slightly inferior results, particularly when models within the ensemble have varying strengths.

Bagging and Boosting, while still strong, exhibit comparatively lower performance. Bagging, which relies on homogeneous base models, struggles to leverage the diversity of model types effectively. Despite its ability to reduce variance through bootstrap aggregation, Bagging does not exploit the significant differences between models, which limits its performance. As a result, its overall predictive power falls short when compared to more diverse methods like Stacking and Voting.

Boosting, although excelling in recall, ranks slightly below both Bagging and Voting in terms of accuracy. Its sequential training process, which focuses on misclassified instances by iteratively building models to correct errors, results in higher computational cost and sensitivity to noisy data. The lack of parallelization in the training phase also contributes to longer training times, further limiting its overall performance in this particular evaluation.

**Figure 11:**
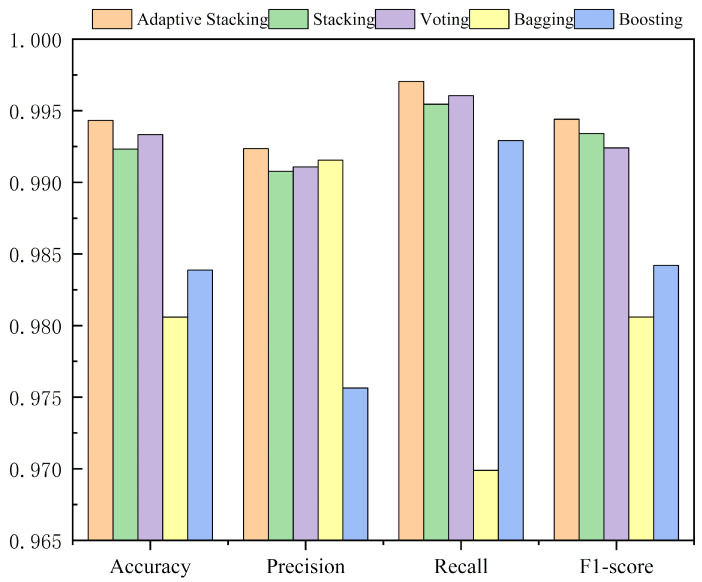
Comparison of integration methods.

In summary, Adaptive Stacking emerges as the most effective ensemble strategy, providing the best balance of accuracy, stability, and robustness. This method’s ability to intelligently combine base learners through a trained meta-model significantly enhances its predictive performance compared to Voting, Bagging, and Boosting. On the other hand, Voting lacks the flexibility to adjust for the varied performance of its base models, making it slightly less effective. Bagging and Boosting, while offering certain advantages, particularly in handling variance and focusing on misclassifications, respectively, do not outperform the more sophisticated ensemble methods like Stacking, as quantitatively evidenced by the near-perfect metrics observed in Figure 11.

### 5.4. Baseline Comparison

To demonstrate the efficacy of MaSS-Droid, we compare its performance against three state-of-the-art (SOTA) baseline methods: FEDroid, HYDRA, and MalScan. FEDroid [34] introduces a federated learning-based framework for Android malware detection, addressing both privacy concerns and malware variant detection. This framework utilizes genetic evolution to simulate malware variants and employs a deep residual network (ResNet) to achieve high detection accuracy. HYDRA [35], on the other hand, is a multimodal deep learning framework for malware classification that integrates multiple feature types, such as API calls, mnemonic sequences, and byte sequences. It employs a modular architecture where each feature type is processed by a dedicated subnetwork, and modality dropout and pretraining techniques are used to enhance generalization and prevent overfitting. MalScan [36] presents a lightweight, graph-based approach for Android malware detection, utilizing social-network-based centrality analysis on function call graphs. By applying various centrality measures, such as degree, closeness, harmonic, and Katz, MalScan effectively detects malware, with particular emphasis on sensitive API calls. A thorough analysis of the experimental results presented in Table 5 indicates that the proposed MaSS-Droid framework outperforms the baseline methods in terms of predictive accuracy, while also effectively mitigating the risks of overfitting. The results demonstrate consistent improvements across key performance metrics, suggesting that MaSS-Droid possesses strong generalization capabilities, which enable it to adapt well to diverse data distributions. Notably, the framework achieves these enhancements in detection accuracy and robustness while maintaining reasonable computational efficiency. The inclusion of time complexity analysis further highlights that MaSS-Droid manages to control processing time effectively, thus balancing high detection performance with practical deployment considerations. These findings substantiate the effectiveness of MaSS-Droid in real-world applications, where both model accuracy and operational feasibility are critical.

**Figure 12:**
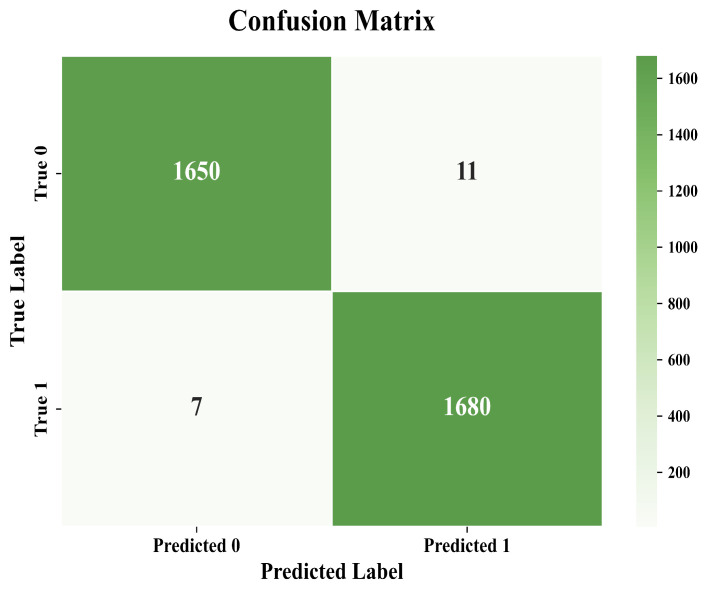
Model evaluation using confusion matrix.

To assess the statistical robustness of the reported performance metrics, we performed 5-fold cross-validation and calculated the 95% confidence intervals (CI) for the F1-Score of each model. The confidence interval provides a range within which the true F1-Score is expected to fall with 95% confidence. The results demonstrate that our proposed model exhibits stable and consistent performance, as indicated by the narrow confidence interval of ±0.001285 for the F1-Score, confirming the reliability of our approach. Figure 12 presents the confusion matrix, which provides a visual representation of the classification performance of the proposed MaSS-Droid model on the test set. The matrix effectively delineates the model’s predictions against the true labels for both malware and benign software categories.As illustrated, the model correctly classifies the vast majority of instances, with the values of TP and TN significantly surpassing those of FP and FN. This distribution demonstrates the model’s exceptional detection accuracy and operational reliability.

### 5.5. Detection Performance Against Emerging Malware

To evaluate the generalization capability and detection performance of the proposed framework MaSS-Droid on a new dataset, we conducted experiments on the CICMalDroid2020 dataset. All thresholds and hyperparameters used in the framework, including those for feature selection and discrimination scoring, were optimized exclusively on the training data from the Drebin and AndroZoo datasets.The feature selection process commenced with variance-based filtering to eliminate low-variance attributes from the initial set of 470 features. A variance threshold of 0.05 was applied, whereby features with variance below this value were discarded. Variance characterizes the degree of fluctuation of a feature across different samples: the lower the variance, the more similar the feature values are among samples, indicating that most instances share nearly identical values on that dimension and thus lack sufficient discriminative power to distinguish between benign and malicious applications. Consequently, such near-constant features were removed, yielding a refined subset of 276 features. This initial dimensionality reduction was achieved efficiently, substantially reducing computational overhead while preserving the essential discriminative characteristics required for subsequent analysis.

**Figure 13:**
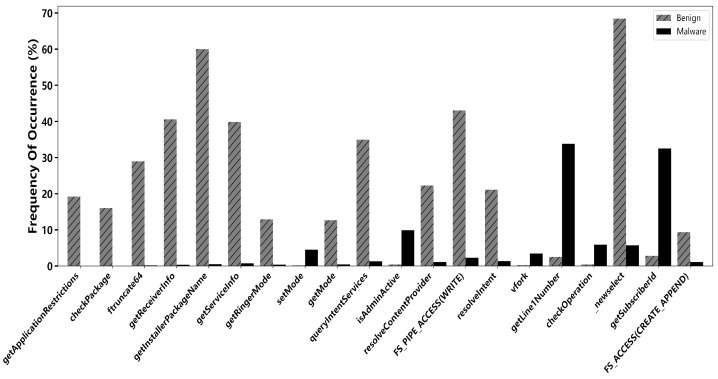
Top 20 feature discrimination score.

Subsequently, the feature discrimination scoring method was employed for the second stage operation of feature refinement. Based on empirical analysis and prior experimental validation, a discrimination threshold of 0.3 was established to retain features exhibiting substantial distributional divergence between malware and benign samples. This step further reduced the feature set from 276 to 65 highly discriminative features. These retained features demonstrate significant differences in their occurrence frequencies across the two classes, underscoring their relevance and potency in characterizing malicious behavior. The results demonstrate significant distributional differences in these features between malicious and benign applications as Figure 13. According to the proposed methodology, a Feature Discrimination Score close to 1 indicates that a feature occurs significantly more frequently in one class than the other, reflecting strong discriminative power. In Table 6, getApplicationRestrictions (Score = 0.9995) appears in 19.22% of benign samples but only 0.01% of malicious samples, demonstrating its high effectiveness in identifying malicious behavior; setMode (Score = 0.9629) is used in 4.51% of malicious samples compared to only 0.17% in benign samples, suggesting its frequent exploitation for sensitive operations such as privilege escalation; and getLineINumber (Score = 0.9259) is invoked in 33.84% of malicious samples versus merely 2.51% in benign samples, further validating the capability of the MaSS-Droid framework in recognizing sensitive behaviors. These highly discriminative features span multiple dimensions, including system calls, permission checks, and service queries, illustrating the comprehensiveness and robustness of the multi-level feature screening mechanism in capturing diverse malicious behaviors and providing reliable feature support for detecting novel and evolving malware.

Finally, by using the FAIG method to select features and setting the threshold at the experimental average, this method integrates the two criteria of feature information gain and occurrence frequency. It selected 21 most discriminative features from the 65 features at the previous level. Table 7 presents the top 10 features selected by FAIG. Unlike conventional information gain approaches, the FAIG methodology considers both the discriminative power of features and the stability of their distributions, effectively mitigating model overfitting caused by feature sparsity. Experimental results demonstrate that this approach reduces feature dimensionality by 66% while maintaining classification performance, significantly enhancing both model generalization capability and computational efficiency. The selected feature set comprehensively covers characteristic behavioral patterns of malware, including system calls, network access, and private data acquisition, establishing a reliable feature engineering foundation for constructing lightweight, high-precision Android malware detection models.

This study employs a multi-level feature screening framework to refine an initial set of 470 high-dimensional features, ultimately deriving a lightweight feature set comprising only 21 core features. Experimental results as Table 8 demonstrate that this method significantly reduces computational complexity while maintaining high detection efficacy. The superiority of the proposed approach is manifested in three primary aspects:

First, the method achieves a breakthrough in feature dimensionality reduction efficiency. The feature dimensionality was drastically compressed from 470 to a mere 21 features, representing a remarkable reduction of 95.5%. This near two-order-of-magnitude reduction fundamentally mitigates the “curse of dimensionality” and establishes a solid foundation for constructing lightweight models.

Second, the screened feature set maintains highly competitive detection performance. Utilizing the refined 21-feature set, multiple classification models demonstrated excellent performance. Specifically, KNN, Logistic Regression, and SVM models all showed significant improvements in comprehensive performance (F1-score) compared to using the original feature set, with the SVM model exhibiting a particularly substantial increase in F1-score from 53.67% to 67.11%. Although tree-based models (Decision Tree, Random Forest) experienced minor and acceptable performance degradation, their accuracy remained at a high level above 95%, fully validating the strong discriminative power and generalization capability of the refined feature set.

Finally, the method yields qualitative improvements in computational efficiency and practical applicability. The 95.5% reduction in feature quantity directly translates to an order-of-magnitude decrease in computational complexity. For parametric models, the number of parameters to be estimated is substantially reduced; for distance-based algorithms such as KNN, the computational burden is significantly alleviated. This dramatically enhances model training and inference speed while reducing storage requirements and energy consumption, making the deployment of real-time, efficient malware detection systems on resource-constrained mobile devices entirely feasible.

In summary, the multi-level feature screening method proposed in this study successfully identifies crucial information within the high-dimensional feature space, achieving an optimal balance between performance and efficiency by retaining only 4.5% of the original features. This research not only validates the principle that “feature quality supersedes quantity” but also provides robust technical support and a viable pathway for deploying lightweight, high-speed, and reliable malware detection solutions in practical environments.

**Figure 14:**
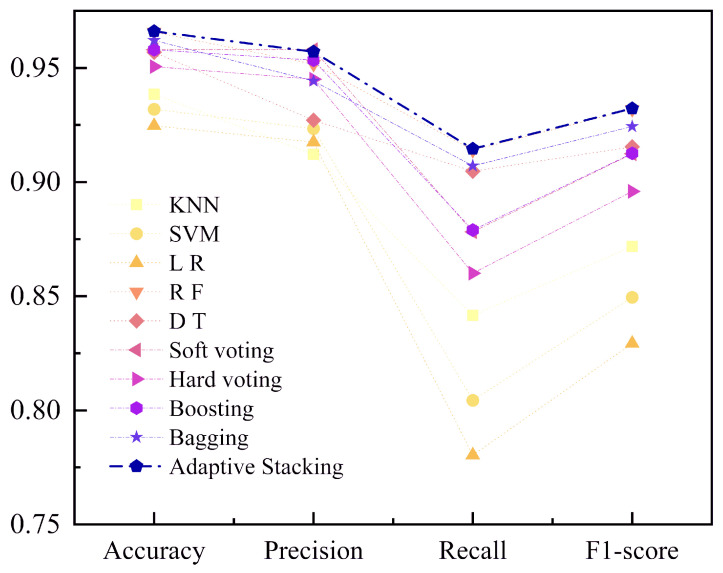
A comparative analysis of the performance between base learners and ensemble models.

As shown in Figure 14, among the compared models, the Stacking ensemble method demonstrated superior performance. By synthesizing the diverse strengths of its constituent models and mitigating their individual biases, it maintained high accuracy while achieving an optimal trade-off between precision and recall. Consequently, the Stacking ensemble delivered exceptional generalization capability and robustness, outperforming all single models and other ensemble techniques.

### 5.6. Quantitative Analysis of Module Ablation

In this ablation study, we systematically evaluated the impact of multi-level feature screening methods on model performance. The first three columns of Table 9 present different feature selection strategies, including Variance Threshold (VT), Feature Discrimination Score (FDS), and Information Gain (FAIG). These screening methods aim to reduce the dimensionality of the input feature set by eliminating redundant or irrelevant features, thereby enhancing the model’s efficiency and computational performance. By sequentially applying these feature selection strategies, irrelevant features are effectively filtered out, leading to improved model performance.

Additionally, we introduced Adaptive Stacking as a key optimization technique. Adaptive Stacking(A-Stacking) aggregates predictions from multiple base models, thereby enhancing the model’s generalization ability and stability. According to the experimental results, the combination of feature screening strategies and adaptive stacking significantly improves model performance across accuracy, precision, recall, and F1-score. Specifically, when all feature selection strategies were combined with adaptive stacking, the final model achieved an accuracy of 96.61% and an F1-score of 92.23%, indicating the critical role that adaptive stacking played in optimizing the model’s performance.

In summary, the experimental results demonstrate that a thoughtful integration of multi-level feature screening and adaptive stacking significantly enhances the predictive performance of the model on complex tasks. The effective combination of these methods provides important insights and practical foundations for model optimization.

## 6. Limitations

This work first validated MaSS-Droid on the Drebin and AndroZoo datasets, where it achieved high performance. Because these datasets were collected in earlier periods and may reflect historical patterns of app behaviour, the near-ideal scores warrant caution: they might in part reflect dataset-specific fitting rather than universal generality. To investigate temporal generalization, we applied the trained model—with all weights and screening thresholds fixed—to CICMalDroid2020 as a temporally separated holdout; this evaluation was performed solely to probe the method’s behaviour on more recent or differently distributed samples and did not involve any retraining or threshold retuning (see Section 5.5). In addition, although provides an initial analysis of algorithmic complexity and an estimate of inference cost, the present study does not include direct, end-to-end measurements of latency, memory use, or energy consumption on representative mobile hardware. As a result, definitive conclusions about on-device deployability and runtime efficiency in resource-constrained settings remain to be established. Finally, while Drebin, AndroZoo and CICMalDroid2020 together provide diverse examples for method development and evaluation, they are not exhaustive of all geographic markets, app stores, or the continuously evolving malware landscape. Future work will therefore prioritize broader cross-temporal and cross-source evaluations, together with empirical on-device profiling, to more comprehensively assess robustness and practical deployability.

## 7. Conclusions

This study presents MaSS-Droid, an Android malware detection framework incorporating multi-feature fusion (permissions, API calls, opcode sequences) and multi-layer feature screening for mitigating feature redundancy. The framework employs variance thresholding, discriminative performance analysis, and frequency-adjusted information gain to iteratively refine features, reducing dimensionality while preserving discriminative power. To address instability limitations in single-model approaches, an adaptive stacked ensemble introduced F1-score-based optimization that dynamically adjusts weights of heterogeneous base classifiers (SVM, KNN, DT, RF, LR) to strengthen robustness against polymorphic malware. Evaluated on 11,160 samples, the framework achieved 99.55% accuracy, outperforming comparative ensemble methods.

Future work will explore several directions to build upon the current foundation. Firstly, to address the performance gap observed with opcode features, we plan to enhance opcode representation by integrating deep sequence models (e.g., BiLSTMs or Transformers). This hybrid approach would leverage deep learning for semantic feature extraction from sequences while retaining the efficient ensemble framework for final classification, thereby combining the strengths of both paradigms. Secondly, we will investigate resolving distance metric degradation in classifiers like KNN through manifold learning techniques (e.g., Isomap, t-SNE) to better preserve feature-space topology after aggressive feature screening. Finally, we will expand into hybrid analysis by incorporating dynamic behavioral features (e.g., system-call traces, network traffic) into the adaptive Stacking architecture to improve detection of evasive malware, concurrently optimizing the entire pipeline for real-world operational efficiency.

## Figures and Tables

**Table 1:** Dataset sources and time ranges.

Type	Dataset	Number	Time Range
Malware	Drebin	5560	Aug. 2010–Oct. 2012
Malware	CICMalDroid2020	10,346	Dec. 2017–Dec. 2018
Benign Software	AndroZoo	5600	Up to Dec. 2022
Benign Software	CICMalDroid2020	1253	Dec. 2017–Dec. 2018

**Table 2:** Comparison of screening effects for API.

Classifier	Data Type	Accuracy	Precision	Recall	F1-Score
L R	Original	0.9905	0.9919	0.9889	0.9900
	Screened	0.9955	0.9959	0.9947	0.9953
SVM	Original	0.9918	0.9928	0.9900	0.9914
	Screened	0.9952	0.9958	0.9941	0.9949
KNN	Original	0.9912	0.9919	0.9887	0.9908
	Screened	0.9914	0.9919	0.9900	0.9909
R F	Original	0.9937	0.9943	0.9924	0.9933
	Screened	0.9951	0.9957	0.9940	0.9948
D T	Original	0.9884	0.9884	0.9873	0.9878
	Screened	0.9896	0.9894	0.9888	0.9891

**Table 3:** Comparison of screening effects for opcode.

Classifier	Data Type	Accuracy	Precision	Recall	F1-Score
L R	Original	0.9809	0.9803	0.9797	0.9800
	Screened	0.9862	0.9855	0.9855	0.9855
SVM	Original	0.9873	0.9881	0.9852	0.9866
	Screened	0.9887	0.9888	0.9875	0.9881
KNN	Original	0.9866	0.9869	0.9850	0.9860
	Screened	0.9877	0.9873	0.9870	0.9871
R F	Original	0.9912	0.9922	0.9895	0.9908
	Screened	0.9920	0.9928	0.9905	0.9916
D T	Original	0.9852	0.9845	0.9845	0.9845
	Screened	0.9848	0.9847	0.9836	0.9841

**Table 4:** Comparison of screening effects for permission.

Classifier	Data Type	Accuracy	Precision	Recall	F1-Score
L R	Original	0.9600	0.9610	0.9550	0.9578
	Screened	0.9709	0.9714	0.9674	0.9693
SVM	Original	0.9655	0.9672	0.9604	0.9635
	Screened	0.9720	0.9727	0.9684	0.9704
KNN	Original	0.9605	0.9615	0.9554	0.9582
	Screened	0.9571	0.9595	0.9504	0.9545
R F	Original	0.9651	0.9663	0.9603	0.9631
	Screened	0.9780	0.9781	0.9756	0.9768
D T	Original	0.9633	0.9645	0.9584	0.9613
	Screened	0.9677	0.9680	0.9642	0.9660

**Table 5:** Performance comparison with baseline methods (with Confidence Intervals and Time Complexity).

Method	Accuracy	F1-Score	Precision	Recall	Time Complexity (Minutes)	CI
Ours	0.9982	0.9988	0.9982	0.9985	2.2 ± 0.2	±0.001285
FEDroid	0.9271	0.9551	0.9551	0.9482	3.5 ± 0.2	±0.002035
HYDRA	0.9517	0.9589	0.9604	0.9556	4.1 ± 0.2	±0.001930
MalScan	0.9612	0.9517	0.9517	0.9432	3.0 ± 0.2	±0.002081

**Table 6:** Features selected by feature discrimination score.

Feature Name	Score ($f_{j}$)	Benign Usage	Malicious Usage
getApplicationRestrictions	0.9995	0.1922	0.0001
checkPackage	0.9987	0.1604	0.0002
ftruncate64	0.9944	0.2897	0.0016
getReceiverInfo	0.9917	0.4061	0.0034
getInstallerPackageName	0.9913	0.6000	0.0052
getServiceInfo	0.9810	0.3983	0.0075
getRingerMode	0.9684	0.1292	0.0041
setMode	0.9629	0.0017	0.0451
getMode	0.9629	0.1265	0.0047
queryIntentServices	0.9626	0.3493	0.0131
isAdminActive	0.9551	0.0045	0.0992
resolveContentProvider	0.9496	0.2228	0.0112
FS_PIPE_ACCESS(WRITE)___	0.9462	0.4306	0.0232
resolveIntent	0.9362	0.2111	0.0135
vfork	0.9361	0.0022	0.0349
getLine1Number	0.9259	0.0251	0.3384
checkOperation	0.9249	0.0045	0.0594
_newselect	0.9164	0.6847	0.0572
getSubscriberId	0.9126	0.0284	0.3250
FS_ACCESS(CREATE__APPEND)__	0.8812	0.0936	0.0111
⋮	⋮	⋮	⋮
FS_PIPE_ACCESS(READ__)_	0.5201	0.0440	0.0917
performDeferredDestroy	0.5121	0.0830	0.0405
addToDisplayWithoutInputChannel	0.5121	0.0830	0.0405
getInstalledPackages	0.5097	0.0808	0.1647
fdatasync	0.5065	0.7889	0.3893

**Table 7:** Top 10 features selected by FAIG.

Feature	FAIG Score
statfs64	0.00040
_newselect	0.00033
fdatasync	0.00028
fchmod	0.00028
NETWORK_ACCESS____	0.00028
getInstallerPackageName	0.00026
pwrite64	0.00025
remove	0.00024
NETWORK_ACCESS()____	0.00024
fchown32	0.00023

**Table 8:** Performance of multi-level feature screening.

Classifier	Features	Dataset	Accuracy	Precision	Recall	F1-Score
L R	470	Original	0.9275	0.9014	0.8049	0.8431
	21	Screened	0.9359	0.9328	0.8131	0.8587
SVM	470	Original	0.8549	0.8234	0.5403	0.5367
	21	Screened	0.8771	0.8391	0.6320	0.6711
KNN	470	Original	0.9160	0.8577	0.8026	0.8264
	21	Screened	0.9334	0.8978	0.8352	0.8623
R F	470	Original	0.9766	0.9734	0.9361	0.9535
	21	Screened	0.9653	0.9534	0.9110	0.9305
D T	470	Original	0.9671	0.9397	0.9341	0.9369
	21	Screened	0.9571	0.9350	0.8963	0.9142

**Table 9:** Quantitative analysis of module ablation.

MFS (Based on KNN)			A-Stacking	Accuracy	Precision	Recall	F1-Score
VT	FDS	FAIG					
×	×	×	×	0.9160	0.8577	0.8026	0.8264
✓	×	×	×	0.9188	0.8642	0.8075	0.8319
✓	✓	×	×	0.9213	0.8645	0.8203	0.8401
✓	✓	✓	×	0.9334	0.8978	0.8352	0.8623
✓	✓	✓	✓	0.9661	0.9571	0.9146	0.9323

## Data Availability

The datasets used in this study are publicly available: the Drebin dataset can be accessed at https://tianchi.aliyun.com/dataset/172774/(accessed on 28 October 2025); Benign applications were collected from the Google Play Store;AndroZoo dataset is available upon request from the AndroZoo project at https://androzoo.uni.lu/(accessed on 28 October 2025); CICMalDroid2020 dataset can be accessed at http://205.174.165.80/CICDataset/MalDroid-2020/ (accessed on 28 October 2025).

## Abbreviations

The following abbreviations are used in this manuscript:

MaSS-Droid	Multi-feature And Multi-layer Screening For Adaptive Stacking Integration
API	Application Programming Interface
APK	Android Application Package
IG	Information Gain
FAIG	Requency Adjusted Information Gain
CI	Confidence Intervals
TP	True Positive
TN	True Negative
FP	False Positive
FN	False Negative
OOF	Out Of Fold
L R	Logistic Regression
SVM	Support Vector Machine
KNN	k-Nearest Neighbors
R F	Random Forest
D T	Decision Tree
MFS	Multi-Level Feature Screening
A-Stacking	Adaptive Stacking
VT	Variance Threshold
FDS	Feature Discrimination Score
